# Retrospective study on the effectiveness of clinical pharmacists’ consultation in the management of carbapenem-resistant organisms infections

**DOI:** 10.3389/fphar.2026.1730591

**Published:** 2026-03-10

**Authors:** Jie Ma, Xiuyan Li, Xiaoyue Liu, Yueming Zhang, Sixi Zhang

**Affiliations:** Department of Clinical Pharmacy, First Hospital of Jinlin University, Changchun, Jilin, China

**Keywords:** antimicrobial stewardship, carbapenem-resistant organisms, clinical pharmacist consultation, retrospective study, treatment outcomes

## Abstract

**Background:**

The emergence and global dissemination of carbapenem-resistant organisms (CROs) pose a significant public health threat. Clinical pharmacists play a pivotal role in optimizing antimicrobial therapy through their expertise in pharmacokinetics, pharmacodynamics, and antimicrobial stewardship.

**Objective:**

This study aimed to assess the impact of clinical pharmacists’ consultations on treatment outcomes in patients with CRO infections.

**Methods:**

A retrospective observational study was conducted using data from patients hospitalized at the First Hospital of Jilin University. Patients who received pharmacist consultations were compared with those who did not. The primary outcomes included clinical improvement and mortality rates.

**Results:**

A total of 285 patients were included. The acceptance rate (AR) of consultation suggestions by clinicians was 89.82%, and the effective response rate (ERR) was 74.03%. Significant differences were observed between the acceptance and non-acceptance groups regarding effective response and mortality. Patients in the non-acceptance group had a lower effective response rate (20.69% vs. 80.08%) and a higher mortality rate (44.80% vs. 9.76%). Multivariate analysis confirmed that acceptance of pharmacists’ recommendations was an independent predictor of effective response (OR = 23.72, 95% CI [7.823–71.917], P < 0.001).

**Conclusion:**

Clinical pharmacists’ consultations significantly improve treatment outcomes in patients with CRO infections. The acceptance of pharmacists’ recommendations is associated with higher effective response rates and lower mortality, underscoring the importance of integrating clinical pharmacists into multidisciplinary teams managing CRO infections.

## 1 Introduction

Carbapenem-resistant organisms (CROs) represent a significant global health threat due to their ability to inactivate most β-lactam antibiotics and frequently exhibit extensive drug resistance, rendering infections challenging to treat (Bonomo et al., 2018; Tamma and Hsu, 2019). According to the Centers for Disease Control and Prevention (CDC), carbapenem-resistant Enterobacteriaceae (CRE) account for approximately 13,100 healthcare-associated infections (HAIs) annually in the United States, including roughly 1,100 deaths, while carbapenem-resistant *Acinetobacter* (CRAB) are associated with about 8,500 HAIs and 700 deaths each year (Antibiotic Resistance Threats in the, 2021). Data from the China Antimicrobial Surveillance Network (CHINET) further indicate a high prevalence of carbapenem-resistant *Klebsiella pneumoniae* in hospital-acquired bloodstream infections (HABSIs) (Hu et al., 2022). In certain regions, carbapenem-resistant *Pseudomonas aeruginosa* (CRPA) infections are also linked to elevated mortality rates (Reyes et al., 2023; Büchler et al., 2023). These findings underscore the urgent need for effective strategies to manage CRO infections, including accurate diagnosis, appropriate antimicrobial selection, and timely surgical interventions such as drainage and debridement.

CRO infections, marked by high mortality, prolonged treatment, and recurrence, cause serious infections in the respiratory tract, urinary tract, and bloodstream, posing major clinical challenges (Müller et al., 2023). Their rising prevalence in diverse healthcare settings underscores a pressing global health issue (Martínez et al., 2023; O'Hara et al., 2025). In response, China implemented antimicrobial stewardship policies, including the “Special Rectification Activities on Clinical Antibiotic Use” (2011–2014), which emphasized the expanding role of infectious diseases clinical pharmacists in optimizing therapy through multidisciplinary collaboration, therapeutic drug monitoring, and regimen review (Qian et al., 2021; Wang et al., 2019; Garau and Bassetti, 2018). While evidence supports the benefits of clinical pharmacist consultation (CPC) in antimicrobial stewardship, its specific impact on CRO infection management remains underexplored (Stuhec, 2021; Zhang et al., 2020; Wang et al., 2020; Zhang et al., 2019). This study therefore aims to evaluate the effectiveness of CPC in improving clinical outcomes for patients with CRO infections.

## 2 Materials and methods

### 2.1 Study design and patients

A retrospective observational study was performed to assess the effectiveness of clinical pharmacists’ consultation in managing carbapenem-resistant organisms infections. Data were collected from patients who were hospitalized at the First Hospital of Jilin University (Changchun, China). The protocol was reviewed and approved by the ethical committee of the hospital (Ethics Approval Number: 2023–531). Due to the retrospective nature of the study, informed consent was waived. From January 2019 to December 2022, a total of 37,916 consultations from antimicrobial clinical pharmacists in the First Hospital of Jilin University have been completed. Of the 37,916 consultations, 327 cases were associated with CROs (0.80%).

Patients were included if they met all of the following criteria:

- Patient hospitalized with a confirmed infection;
- The carbapenem-resistant organism was detected as positive in the all clinically relevant liquid specimens collected for microbiological culture;
- The treating clinician requested a formal CPC specifically for reviewing and adjusting the antimicrobial treatment regimen;
- The CPC was conducted at least 72 h before patient discharge or death to allow for adequate evaluation of treatment efficacy.

Patients were excluded for any of the following reasons:

- The consultation was requested solely for approval of a specific antibiotic (e.g., carbapenem, tigecycline) that had already been initiated, rather than for comprehensive regimen review or adjustment;
- Within 2 days of the CPC, the patient died or was discharged from the hospital, making it impossible to evaluate the efficacy of the drug treatment;
- Incomplete clinical data referred to cases with: a) missing susceptibility results for the causative carbapenem-resistant organism; b) undocumented details of the anti-infective regimen before or after consultation; or c) unavailable data required to assess the primary clinical or microbiological outcomes.

Based on the above criteria, 285 cases were included in this study. To minimize selection bias, consecutive patients receiving CPC were screened, and pre-defined objective inclusion/exclusion criteria were applied uniformly.

### 2.2 Clinical pharmacist consultation

The CPC service is performed by clinical pharmacists specializing in infectious diseases and antimicrobial stewardship, who are core members of the hospital’s multidisciplinary infection control team. The CPC service generally consists of four steps: (1) The clinician sends a consultation request to the Clinical Pharmacy Department when they encounter a problem related to antimicrobial drugs; (2) The Clinical Pharmacy Department assigns an anti-infection clinical pharmacist to the ward to understand the situation of patients; (3) The antimicrobial clinical pharmacist gives drug treatment recommendations according to the site of infection, the severity of infection, the results of bacterial culture, and the benefits and risks of medication; (4) The clinician makes the final decision on treatment.

### 2.3 Definitions

Immunocompromised status was defined as the presence of any of the following conditions or treatments: 1) underlying diseases known to cause immunodeficiency (including active malignancy or malignancy within the past 5 years, human immunodeficiency virus infection/AIDS, history of solid organ or hematopoietic stem cell transplantation, autoimmune diseases, or primary immunodeficiency disorders); or 2) the ongoing use of systemic immunosuppressive therapy within 30 days prior to the index infection. Immunosuppressive therapy included chemotherapy, high-dose corticosteroids (prednisone ≥20 mg/day or equivalent for ≥2 weeks), biologic agents, or other immunosuppressants (e.g., calcineurin inhibitors, antimetabolites).

Acceptance of consultation recommendation: Defined as the clinician’s subsequent prescription being fully aligned with the pharmacist’s core suggestions regarding the selection, dosing, or duration of antimicrobial agents. Acceptance rate (AR) was calculated as the number of consultation recommendations accepted divided by the total number of recommendations provided, expressed as a percentage.

The primary efficacy outcome was the effective clinical response, a composite endpoint assessed at the first comprehensive evaluation after completion of the CPC-guided therapy (or at least 3 days post-CPC). It was categorized as complete response or partial response.

Complete response (CR) required meeting all of the following criteria:

- Clinical resolution: Complete disappearance of all local and systemic symptoms/signs attributable to the index infection (e.g., fever, purulent sputum, dysuria), with stable vital signs.
- Laboratory improvement: Key infection biomarkers (e.g., procalcitonin, C-reactive protein) returned to within normal institutional reference ranges.
- Microbiological eradication (if applicable): Follow-up culture from the original site of infection was negative.
- Radiological resolution (if applicable): Significant resolution or complete absorption of infiltrates/abscesses on follow-up imaging.

Partial Response (PR) defined as not meeting all criteria for CR, but demonstrating significant improvement as evidenced by at least one of the following:

- Marked clinical improvement: Substantial but incomplete resolution of symptoms/signs. For fever, this was strictly defined as a reduction in peak body temperature by ≥ 1.0 °C and decreased fever frequency. For other symptoms (e.g., sputum volume, dysuria), improvement was based on documented clinician assessment and description in the medical record.
- Significant laboratory improvement: A ≥50% reduction from baseline in primary infection biomarkers.
- Radiological improvement: ≥30% reduction in the size of infiltrates/abscesses on imaging.

Treatment failure included lack of improvement, worsening of symptoms/signs, death due to infection, or need for a major change in antimicrobial therapy due to lack of efficacy. The Effective Response Rate (ERR) was calculated as the proportion of patients achieving either a Complete or Partial Response among the total evaluable patients.

To ensure objectivity and reproducibility, outcome assessment was standardized using the explicit criteria described above. Importantly, the two researchers responsible for evaluating clinical outcomes were blinded to the clinicians’ acceptance status (i.e., whether the patient was in the Acceptance or Non-Acceptance group) during the outcome adjudication process. This blinding helps mitigate potential assessment bias.

### 2.4 Statistical analysis

Continuous variables with non-normal distribution are represented as median (Q1-Q3) and compared using the Mann–Whitney U test. Categorical variables are represented as frequency (percentage) and compared using the Chi-square or Fisher’s exact test. For binary outcomes, independent predictors were identified using multivariable logistic regression. For continuous outcomes, linear regression was used. Results are reported as adjusted coefficients (β) with 95% CIs. Results are reported as adjusted odds ratios (aORs) with 95% CIs. All of the analyses were conducted with the SPSS program (version 18.0, Chicago, IL, USA), and *P* values <0.05 were considered to be statistically significant.

## 3 Results

### 3.1 Baseline characteristics of the study population

A total of 285 patients with confirmed CRO infections were included in the final analysis and subsequently divided into two groups: the Acceptance Group (n = 256, 89.8%) and the Non-Acceptance Group (n = 29, 10.2%). The baseline demographic and clinical characteristics of the total cohort and the comparative analysis between groups are summarized in Table 1. The overall study population was predominantly male (72.7%) with a mean age of 59.7 ± 16.6 years. Age distribution across predefined categories showed the highest proportion in the 41–65 years range (49.7%). Clinically, a majority of patients had at least one underlying disease (68.9%), with 25.2% having more than three comorbidities. Nearly half of the patients (49.0%) underwent surgery prior to infection, and a significant history of broad-spectrum antibiotic exposure (79.4%) was common. Immunodeficiency factors were present in 26.6% of patients, and 51.7% required mechanical ventilation. In terms of infection severity, 24.5% of patients were admitted to the intensive care unit (ICU), 16.1% presented with septic shock, and median levels of C-reactive protein (CRP) and procalcitonin (PCT) were 56.3 mg/L (IQR: 28.4–124.1) and 0.47 ng/mL (IQR: 0.21–2.56), respectively. Regarding pathogen distribution, CRAB was the most prevalent (49.3%), followed by CRPA (23.6%) and Carbapenem-resistant *K. pneumoniae* (CRKP, 22.2%). The primary site of infection was the respiratory system (72.7%), followed by intra-abdominal infections (12.6%). Comparative analysis revealed no statistically significant differences (p > 0.05) between the Acceptance and Non-Acceptance groups across all measured baseline characteristics, including demographics, clinical features, pathogen types, and infection sites. This indicates that the two groups were well-balanced and comparable at baseline, suggesting that subsequent differences in treatment outcomes are less likely to be attributable to confounding by these initial patient characteristics.

**TABLE 1:** Baseline characteristics of the study population (N = 286).

Characteristic	Overall (n = 286)	Acceptance group (n = 257)	Non-acceptance group (n = 29)	P value	Statistical test
Gender	​	​	​	0.382	Pearson’s chi-square test
Male	208 (72.7)	189 (73.5)	19 (65.5)	​	​
Female	78 (27.3)	68 (26.5)	10 (34.5)	​	​
Age (years)	59.7 ± 16.6	59.4 ± 16.4	62.8 ± 19.0	0.291	Independent t-test
Age group	​	​	​	0.529	Pearson’s chi-square test
<18	4 (1.4)	3 (1.2)	1 (3.4)	​	​
18–40	30 (10.5)	28 (10.9)	2 (6.9)	​	​
41–65	142 (49.7)	130 (50.6)	12 (41.4)	​	​
66–80	78 (27.3)	67 (26.1)	11 (37.9)	​	​
>80	32 (11.2)	29 (11.3)	3 (10.3)	​	​
Presence of underlying disease	197 (68.9)	175 (68.1)	22 (75.9)	0.392	Pearson’s chi-square test
Number of underlying diseases	​	​	​	0.198	Pearson’s chi-square test
1	89 (31.1)	82 (31.9)	7 (24.1)	​	​
2–3	91 (31.8)	78 (30.4)	13 (44.8)	​	​
>3	72 (25.2)	68 (26.5)	4 (13.8)	​	​
3	34 (11.9)	29 (11.3)	5 (17.2)	​	​
Previous surgery before infection	140 (49.0)	124 (48.2)	16 (55.2)	0.480	Pearson’s chi-square test
History of broad-spectrum antibiotic exposure	227 (79.4)	204 (79.4)	23 (79.3)	0.993	Pearson’s chi-square test
Immunodeficiency factors	76 (26.6)	68 (26.5)	8 (27.6)	0.896	Pearson’s chi-square test
Mechanical ventilation	148 (51.7)	132 (51.4)	16 (55.2)	0.697	Pearson’s chi-square test
ICU admission	70 (24.5)	63 (24.5)	7 (24.1)	0.964	Pearson’s chi-square test
CRP level	56.3 (28.4–124.1)	55.03 (28.4–124.1)	83.5 (26.9–162.2)	0.523	Rank-sum test
PCT level	0.47 (0.21–2.56)	0.46 (0.21–2.16)	1.13 (0.12–4.44)	0.493	Rank-sum test
Presence of septic shock	46 (16.1)	39 (15.2)	7 (24.1)	0.282	Fisher’s exact test
Multidrug-resistant organism	​	​	​	0.344	Pearson’s chi-square test
CRKP	63 (22.2)	52 (20.4)	11 (37.9)	​	​
CRPA	67 (23.6)	60 (23.5)	7 (24.1)	​	​
CRAB	140 (49.3)	130 (51.0)	10 (34.5)	​	​
CR *Escherichia coli*	3 (1.1)	3 (1.2)	0 (0.0)	​	​
CR *Enterobacter cloacae*	9 (3.2)	8 (3.1)	1 (3.4)	​	​
Raoultella planticola	2 (0.7)	2 (0.8)	0 (0.0)	​	​
Infection site	​	​	​	0.109	Pearson’s chi-square test
Pulmonary	208 (72.7)	188 (73.2)	20 (69.0)	​	​
Abdominal	36 (12.6)	28 (10.9)	8 (27.6)	​	​
Intracranial	17 (5.9)	16 (6.2)	1 (3.4)	​	​
Bloodstream	3 (1.0)	3 (1.2)	0 (0.0)	​	​
Skin and soft tissue	12 (4.2)	12 (4.7)	0 (0.0)	​	​
Urinary tract	10 (3.5)	10 (3.9)	0 (0.0)	​	​

### 3.2 Infection sites and pathogen distribution

The most frequent site of infection was the respiratory system (72.98%, n = 208), followed by intra-abdominal infections (12.63%, n = 36) and infections of the central nervous system (5.96%, n = 17). Sputum was the most common specimen yielding a positive culture (65.96%, n = 188). The predominant carbapenem-resistant pathogens isolated were *Acinetobacter baumannii* (48.8%, n = 139), *Pseudomonas aeruginosa* (24.9%, n = 71), and *Klebsiella pneumoniae* (21.4%, n = 61) (Table 1).

### 3.3 Impact of recommendation acceptance on treatment efficacy and mortality

The overall acceptance rate (AR) of the clinical pharmacists’ consultation recommendations by treating clinicians was 89.82% (256/285). For the entire cohort, the effective response rate (ERR) was 74.03% (211/285). A comparative analysis of outcomes between patients whose physicians accepted the consultation advice (Acceptance group, n = 256) and those whose physicians did not (Non-acceptance group, n = 29) revealed significant disparities. As detailed in Table 2, the effective response rate was substantially higher in the acceptance group (80.08% vs. 20.69%, *P* < 0.001). Concordantly, all-cause mortality was significantly lower in the acceptance group (9.76% vs. 44.8%, *P* < 0.001).

**TABLE 2:** Drug treatment results between the acceptance and nonacceptance groups.

Drug treatment results	Acceptance group (n = 256)	Nonacceptance group (n = 29)	*χ2*	*P* value
Effective response (%)	205 (80.08%)	6 (20.69%)	47.794	<0.001
Ineffective response (%)	51 (19.92%)	23 (79.31%)		
Mortality (%)	25 (9.76%)	13 (44.8%)	20.994	<0.001

Data are expressed as the median [IQR] and number (percentage). Differences in the values of categorical variables were compared using the Chi-square test or Fisher’s exact test.

### 3.4 Multivariate analysis of factors predicting effective response

To identify independent predictors of a positive treatment outcome, a multivariate logistic regression analysis was performed, adjusting for potential confounders including age, comorbidities, infection site, and pathogen species. The results, presented in Table 3, demonstrated that acceptance of the clinical pharmacist’s consultation advice was the strongest independent factor associated with achieving an effective clinical response (Odds Ratio [OR] = 23.72, 95% Confidence Interval [CI]: 7.82–71.92, *P* < 0.001).

**TABLE 3:** Multivariate analyses of predictive risk factors for effective response in patients with CRO infections.

Variables	OR	95% Cl	*P* value
Age	1.082	0.725–1.616	0.698
With comorbidity (Y/N)	0.342	0.147–0.798	0.013
With operation history within 1 month prior to infection (Y/N)	1.753	0.820–3.747	0.147
With history of exposure to broad-spectrum antibiotics for more than 2 weeks within 3 months prior to infection (Y/N)	0.758	0.305–1.879	0.549
With immunocompromised host factors (Y/N)	1.110	0.513–2.401	0.791
With mechanical ventilation (Y/N)	0.170	0.075–0.387	<0.001
Microorganism			
*Klebsiella pneumoniae*	1.00	—	—
*Escherichia coli*	0.971	0.320–2.942	0.958
*Enterobacter cloacae*	0.609	0.237–1.565	0.303
*Raoultella planticola*	0.344	0.450–2.215	0.999
*Pseudomonas aeruginosa*	0.189	0.029–1.220	0.080
*Acinetobacter baumannii*	0.044	0.002–0.901	0.043
Infectious site			
Respiratory system	1.00	—	—
Intra-abdomen	0.404	0.141–1.160	0.092
Central nervous system	0.050	0.011–0.222	<0.001
Bloodstream	0.103	0.007–1.542	0.100
Skin or soft tissue	0.620	0.131–2.922	0.545
Urogenital system	0.866	0.052–14.371	0.920
Consultation advice accepted(Y/N)	23.720	7.823–71.917	<0.001

OR, odds ratio; CI, confidence interval; Y/N, yes or no.

### 3.5 Distribution of consultation requests across clinical departments

**FIGURE 1:**
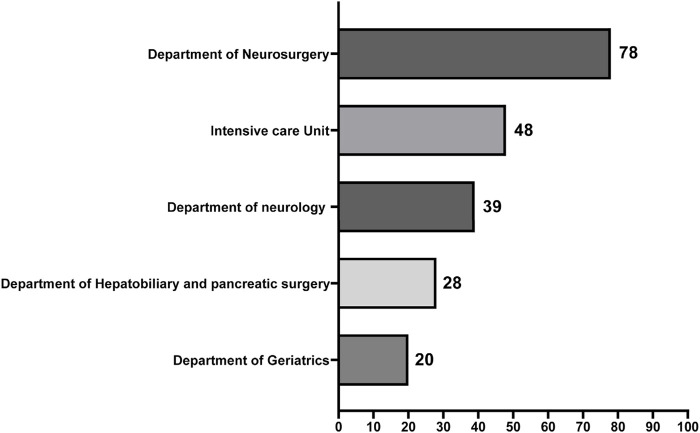
Distribution of top five consultation departments of clinical pharmacists. The horizontal coordinate represents the number of consultation cases, and the vertical coordinate represents the consultation departments.

Consultation requests were received from 25 different clinical departments, reflecting the widespread challenge of CRO infections. The top five departments with the highest number of consultation requests were neurosurgery, intensive care medicine (ICU), neurology, hepatobiliary and pancreatic surgery, and geriatrics (Figure 1).

## 4 Discussion

This retrospective study of 285 patients with carbapenem-resistant organism (CRO) infections demonstrates the critical role of clinical pharmacist consultations in optimizing antimicrobial therapy and improving patient outcomes. The principal finding is that a high clinician AR of pharmacists’ recommendations was strongly associated with a higher ERR and a reduced mortality rate. Multivariate analysis confirmed that acceptance of the pharmacist’s consultation advice was an independent predictor of effective response, aligning with prior stewardship research (Fels, 2020; Xia et al., 2024; Yu et al., 2023). Notably, the non-acceptance group in our study—representing cases where clinicians chose an alternative regimen after considering the pharmacist’s advice—exhibited markedly poorer outcomes. This real-world dynamic underscores that the pharmacist’s input is not merely adjunctive but an active therapeutic component. The large effect size (OR > 20) further supports the formal integration of clinical pharmacist consultation into standard care pathways for CRO infections.

The high AR of 89.82% observed in this study exceeds rates reported in several similar studies, which have typically ranged from 75% to 84% (Felgueiras, 2022; Miocinovic et al., 2015; Van Hoang et al., 2021). This high rate reflects a strong level of trust and confidence from clinicians in the expertise of infectious diseases-specialized clinical pharmacists. This acceptance also likely stems from the highly individualized nature of the recommendations, which integrate multiple factors including infection site, pathogen susceptibility, renal function, pharmacokinetic/pharmacodynamic principles, and potential drug interactions. Furthermore, the clinical pharmacists in our institution are embedded as core, voting members of the formal infectious diseases multidisciplinary team. This structural integration involves their routine participation in daily or weekly patient care rounds and case conferences. Within this framework, they have real-time access to electronic medical records, microbiology results, and bedside clinical discussions. This privileged position allows them to formulate recommendations that are not only evidence-based but also immediately responsive to the evolving clinical context and seamlessly aligned with the overall care plan formulated by the team, thereby ensuring the recommendations are both timely and context-specific.

Consultation requests originated from 25 different clinical departments, with the highest demand coming from units managing critically ill and complex patients, such as neurosurgery, intensive care (ICU), and neurology. This underscores that CRO infections represent a hospital-wide challenge, not limited to traditional infectious diseases or pulmonary departments. The most commonly isolated pathogens, carbapenem-resistant CRAB and *Pseudomonas aeruginosa* (CRPA), are consistent with data from major Chinese resistance surveillance networks like CHINET (Qin et al., 2024; Cui et al., 2022), indicating that our study population is representative of the current epidemiological landscape.

The findings of this study provide a foundation for future research. The single-center, retrospective cohort offers a robust real-world snapshot but warrants validation in broader, prospective settings. The clinical endpoint of “effective response,” though based on explicit criteria, underscores the need for further standardized outcomes in stewardship research. Most importantly, despite multivariable adjustment for available confounders, the possibility of residual confounding cannot be excluded. For instance, clinicians who chose to accept pharmacist recommendations may have differed systematically from those who did not in unmeasured ways, such as their overall practice style, level of engagement in antimicrobial stewardship, or the perceived complexity of the case. It is also possible that patients in the non-acceptance group had subtle, undocumented clinical characteristics (e.g., higher frailty, greater hemodynamic instability) that influenced both the clinician’s decision and the ultimate outcome. These unmeasured factors could partially account for the observed association between recommendation acceptance and improved outcomes. Consequently, future studies with more comprehensive data collection and prospective designs are needed to minimize such bias and to establish causality more definitively.

## 5 Conclusion

In conclusion, this study demonstrates that clinical pharmacists, through their specialized consultation services, play an indispensable role in the management of CRO infections. High clinician adherence to pharmacist recommendations is directly associated with improved survival and significantly higher treatment success rates. The deep integration of clinical pharmacists into multidisciplinary teams is an effective and essential strategy for optimizing antimicrobial use and addressing the global challenge of antimicrobial resistance. Prospective, multi-center studies are encouraged to further validate these findings and to delineate the most cost-effective models for pharmacist intervention.

## Data Availability

The raw data supporting the conclusions of this article will be made available by the authors, without undue reservation.
